# Supplementary material for: Protein buffering of aneuploidy is driven by coordinated factors identified through machine learning
Source: Mol Syst Biol. 2026 Jan 22;22(4):563–98. doi: 10.1038/s44320-026-00187-9 (PMC13046899; doi:10.1038/s44320-026-00187-9)
Supplement: Supplementary file 12 — Expanded View Figures [file 44320_2026_187_MOESM12_ESM.pdf]

Expanded View Figures

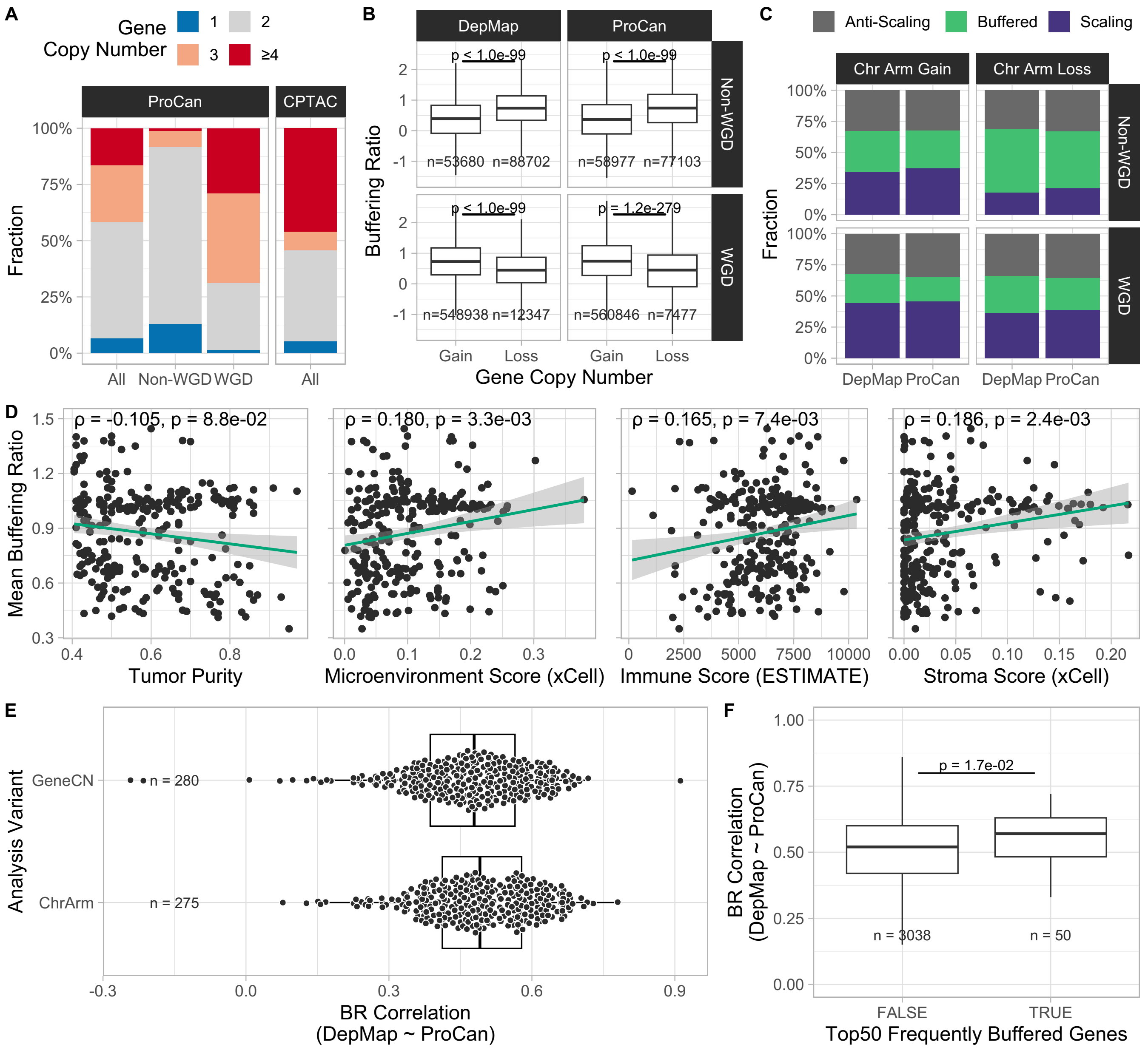

Figure EV1. Controlling for whole-genome doubling status and tumor purity confirms observed protein buffering patterns.

(**A**) Distribution of gene copy numbers among cell lines (ProCan) and tumor samples (CPTAC) separated by their whole-genome doubling (WGD) status. (**B**) Difference in buffering ratio distribution of all proteins between gene copy number gain and loss separated by the WGD status. (**C**) Categorical distribution of buffering classes across pan-cancer cell line datasets upon gene copy number gain and loss separated by WGD status. (**D**) Correlation between mean buffering ratio per tumor sample (CPTAC) and purity scores obtained from metadata provided by CPTAC (Spearman's ρ). (**E**) Correlation of buffering ratios per cell line between DepMap and ProCan (Spearman's ρ), separated by buffering ratios created using either gene (GeneCN) or chromosome arm copy numbers (ChrArm). (**F**) Gene-wise buffering ratio correlation between DepMap and ProCan (GeneCN, Spearman's ρ), separated by their presence in the list of frequently buffered genes with low BR variance. (**B**, **E**, **F**) Boxes represent the interquartile range (IQR) with the central line indicating the median. The whiskers extend to the data points within 1.5×IQR. *P* values were determined using a two-tailed Wilcoxon rank-sum (Mann–Whitney *U*) test.

© The Author(s)

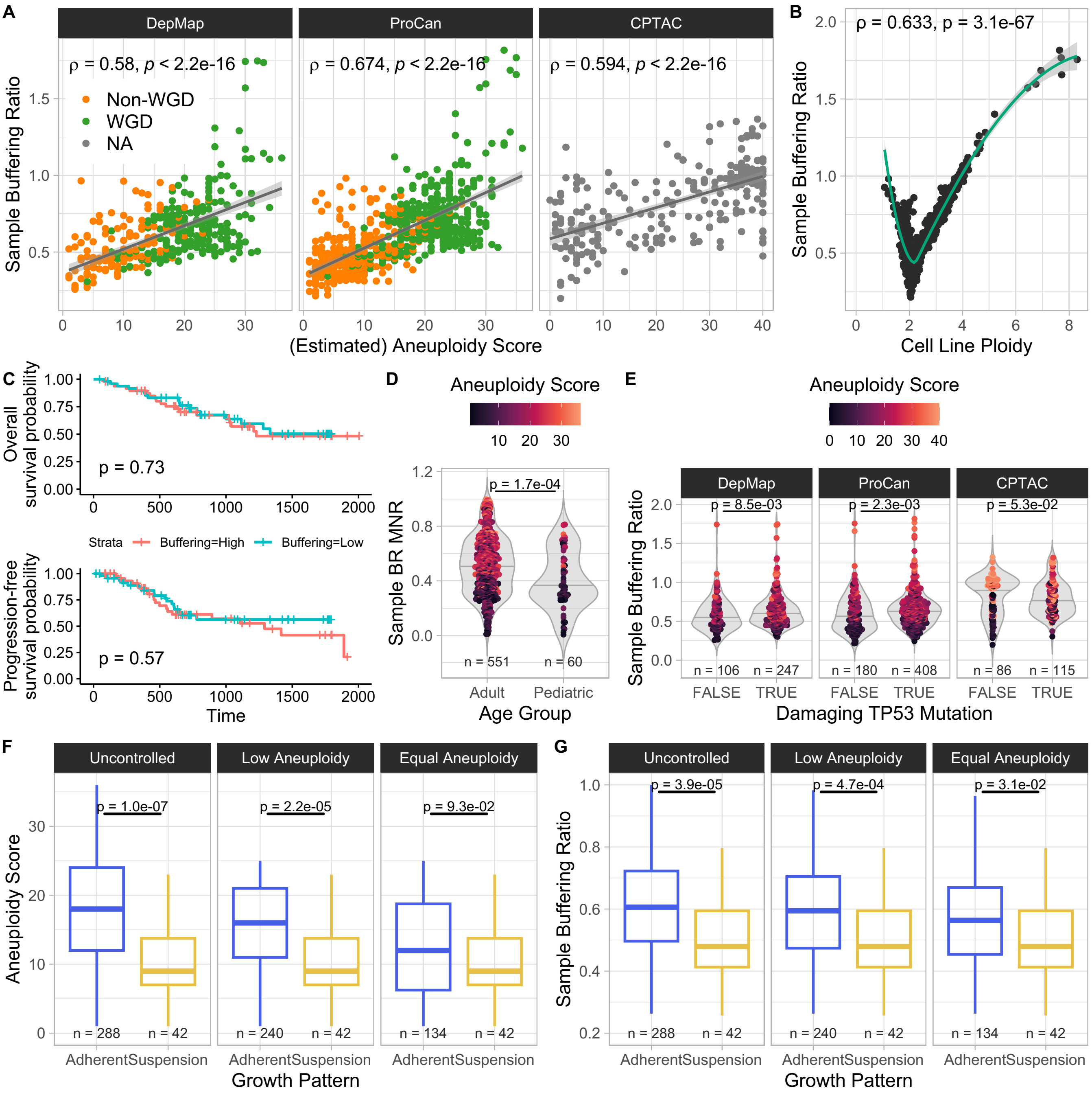

Figure EV2. Average buffering per sample shows relationship with cellular and clinical features of cancer.

(**A**) Scatter plot showing a positive correlation between sample buffering ratio and aneuploidy score in all pan-cancer datasets (Spearman's ρ). (**B**) Scatter plot showing a LOESS-smoothed non-linear relationship between sample buffering ratio and cell line ploidy (ProCan; Spearman's ρ). (**C**) Kaplan–Meier plots showing a decreased progression-free and overall survival probability for tumor samples with high buffering (>80% quantile of sample buffering ratio) compared to low-buffering (<20% quantile, CPTAC). (**D**) Difference in sample buffering ratio mean normalized ranks between age categories shows decreased sample-wide buffering in pediatric cancers (Wilcoxon rank-sum test). (**E**) Difference in sample buffering ratio between samples with and without damaging TP53 mutations (Wilcoxon rank-sum test). (**F**) Difference in aneuploidy between cell lines grown in suspension and adherent cell culture types when applying aneuploidy control methods (DepMap). Controlled for aneuploidy by removing cell lines from the adherent cohort with aneuploidy scores above the maximum of the suspension cohort (middle), and by using stratified sampling to ensure an equal distribution of aneuploidy scores (right). (**G**) Difference in sample buffering ratio between cell lines grown in suspension and adherent cell culture types, controlled for aneuploidy (DepMap). (**F**, **G**) Boxes represent the interquartile range (IQR) with the central line indicating the median. The whiskers extend to the data points within 1.5×IQR. (**D**–**G**) *P* values were determined using a two-tailed Wilcoxon rank-sum (Mann–Whitney *U*) test.

 © The Author(s)

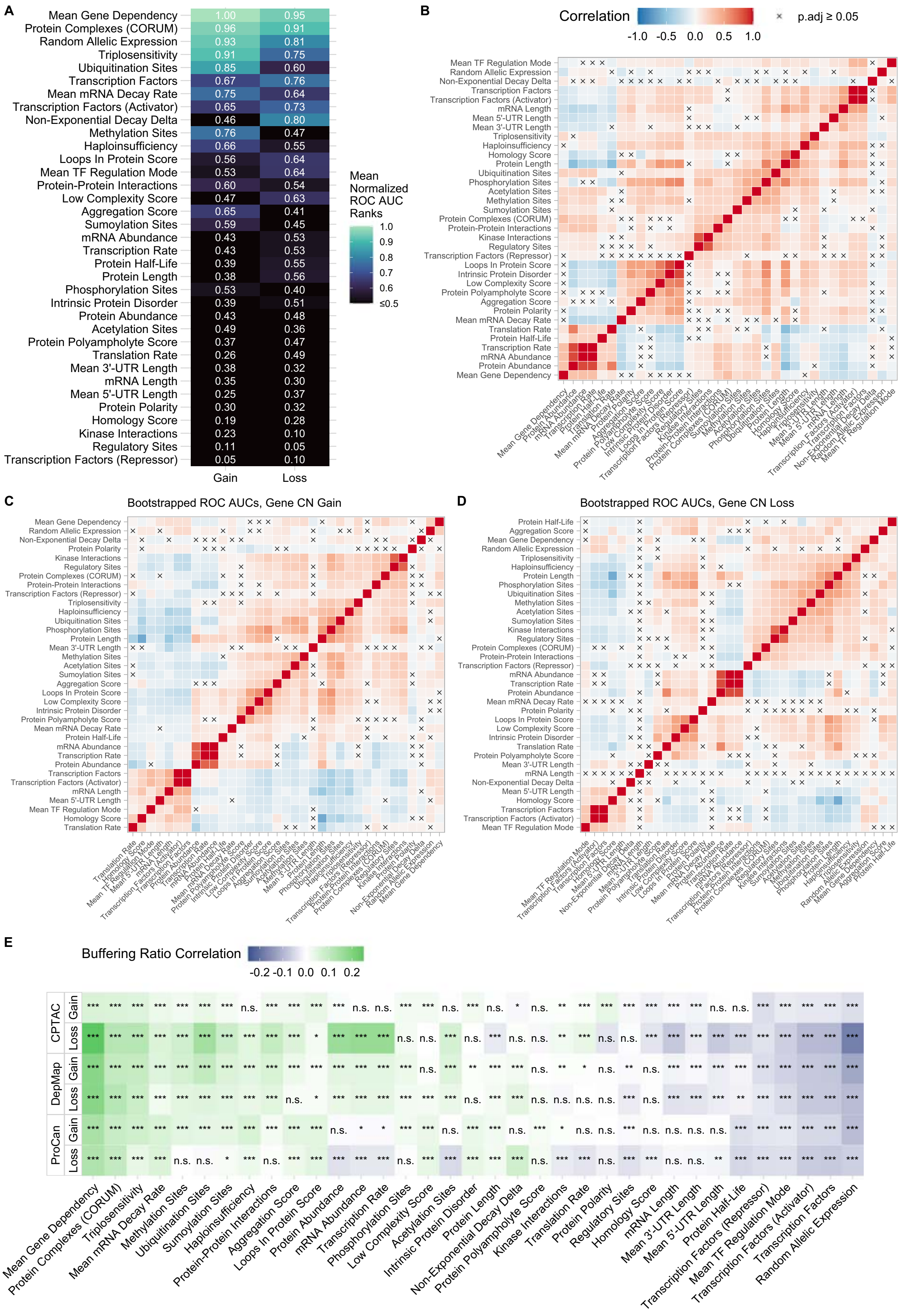
A
Mean Gene Dependency 1.00 0.95
Protein Complexes (CORUM) 0.96 0.91
Random Allelic Expression 0.93 0.81
Triplosensitivity 0.91 0.75
Ubiquitination Sites 0.85 0.60
Transcription Factors 0.67 0.76
Mean mRNA Decay Rate 0.75 0.64
Transcription Factors (Activator) 0.65 0.73
Non-Exponential Decay Delta 0.46 0.80
Methylation Sites 0.76 0.47
Haploinsufficiency 0.66 0.55
Loops In Protein Score 0.56 0.64
Mean TF Regulation Mode 0.53 0.64
Protein-Protein Interactions 0.60 0.54
Low Complexity Score 0.47 0.63
Aggregation Score 0.65 0.41
Sumoylation Sites 0.59 0.45
mRNA Abundance 0.43 0.53
Transcription Rate 0.43 0.53
Protein Half-Life 0.39 0.55
Protein Length 0.38 0.56
Phosphorylation Sites 0.53 0.40
Intrinsic Protein Disorder 0.39 0.51
Protein Abundance 0.43 0.48
Acetylation Sites 0.49 0.36
Protein Polyampholyte Score 0.37 0.47
Translation Rate 0.26 0.49
Mean 3'-UTR Length 0.38 0.32
mRNA Length 0.35 0.30
Mean 5'-UTR Length 0.25 0.37
Protein Polarity 0.30 0.32
Homology Score 0.19 0.28
Kinase Interactions 0.23 0.10
Regulatory Sites 0.11 0.05
Transcription Factors (Repressor) 0.05 0.10
Gain Loss
Mean Normalized ROC AUC Ranks
1.0 0.9 0.8 0.7 0.6 ≤0.5
B
Correlation
-1.0 -0.5 0.0 0.5 1.0
p.adj ≥ 0.05
C
Bootstrapped ROC AUCs, Gene CN Gain
D
Bootstrapped ROC AUCs, Gene CN Loss
E
Buffering Ratio Correlation
-0.2 -0.1 0.0 0.1 0.2
CPTAC Gain
CPTAC Loss
DepMap Gain
DepMap Loss
ProCan Gain
ProCan Loss

Figure EV3. Correlation patterns of potential dosage compensation factors.

(**A**) Mean normalized ranks (MNRs) of ROC AUCs of each factor used for classifying whether a gene is *Buffered* on protein level or *Scaling*, while excluding the *Anti-Scaling* class. MNRs were calculated across all analysis variants (GeneCN, ChrArm, ChrArmAvg) and pan-cancer datasets (DepMap, ProCan, CPTAC), grouped by gene or chromosome arm copy number gain and loss. WGD-controlled subsets were excluded. (**B–D**) Clustered heatmaps showing the correlation (Spearman's ρ) between factors. Correlations with insignificant Benjamini–Hochberg adjusted *P* values were crossed out ($p_{adj} \geq 0.05$). Correlations were calculated using factor values (**B**), bootstrapped ROC AUCs for predicting protein buffering upon gene copy number gain (**C**), and bootstrapped ROC AUCs upon gene copy number loss (**D**). (**E**) Spearman correlation between factors values and buffering ratios upon gene copy number loss and gain across all pan-cancer datasets (DepMap, ProCan, CPTAC).

 © The Author(s)

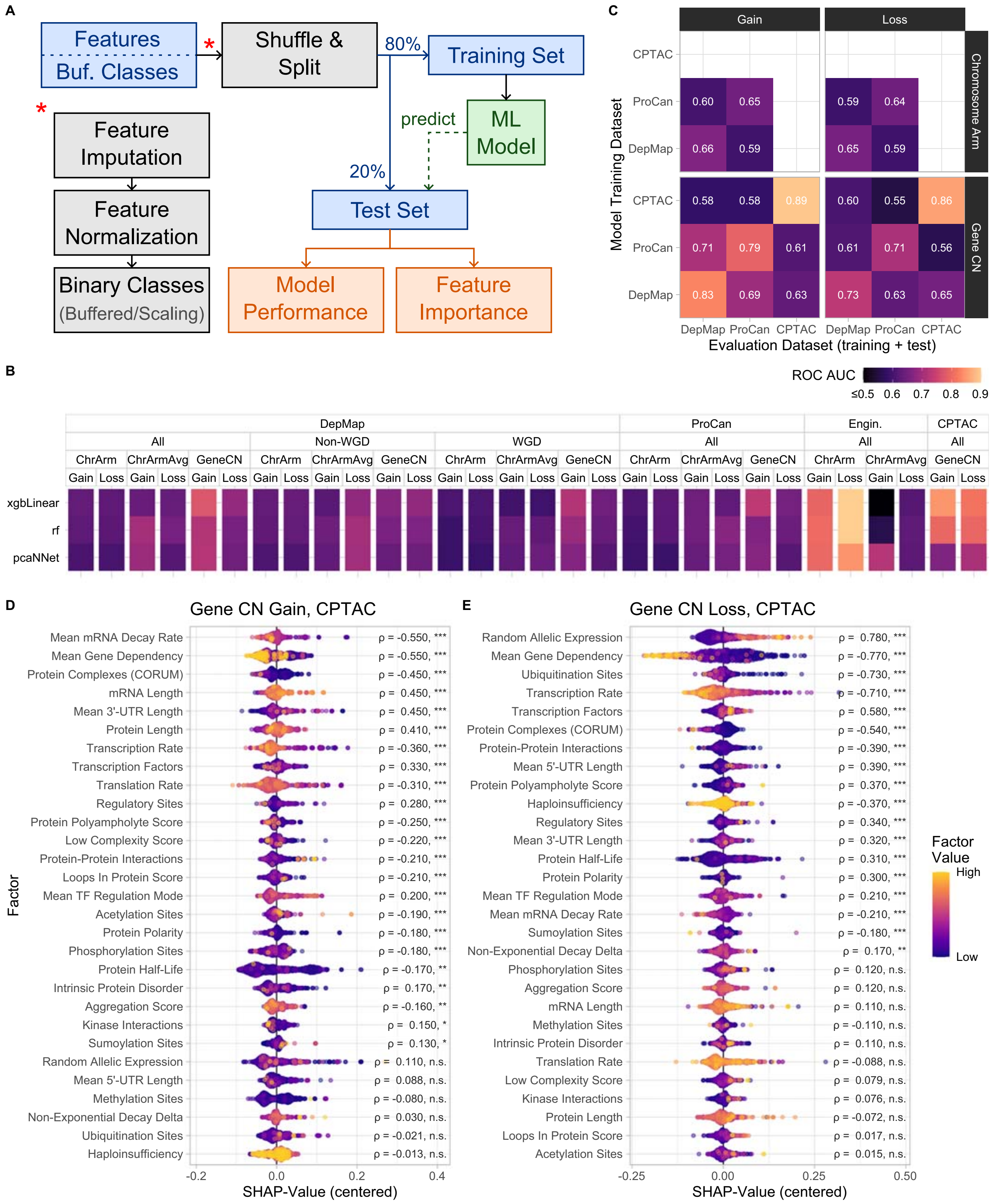
A
Features
Buf. Classes
Shuffle & Split
80%
Training Set
ML Model
predict
20%
Test Set
Feature Imputation
Feature Normalization
Binary Classes (Buffered/Scaling)
Model Performance
Feature Importance
B
ROC AUC
≤0.5 0.6 0.7 0.8 0.9
DepMap
ProCan
Engin.
CPTAC
All
Non-WGD
WGD
ChrArm ChrArmAvg GeneCN
Gain Loss
xgbLinear
rf
pcaNNet
C
Gain
Loss
Chromosome Arm
Gene CN
Model Training Dataset
CPTAC
ProCan
DepMap
Evaluation Dataset (training + test)
D
Gene CN Gain, CPTAC
E
Gene CN Loss, CPTAC
Factor
SHAP-Value (centered)
Factor Value
High
Low

Figure EV4. Multifactorial models improve prediction performance of protein buffering.

(**A**) Illustration of training and evaluation procedures of multifactorial models. (**B**) Model performance (ROC AUC) of trained models, separated by model architecture (xgbLinear, rf, pcaNNet), dataset (DepMap, ProCan, CPTAC, Engineered), subset (All, WGD, Non-WGD), analysis variant (GeneCN, ChrArm, ChrArmAvg), and copy number event (Gain, Loss). (**C**) Out-of-sample performance of multifactorial models trained on buffering classes derived from gene and chromosome arm copy number gain and loss data from different pan-cancer datasets (DepMap, ProCan, CPTAC). Model performance was evaluated using the ROC AUC of model predictions on merged training and test datasets. (**D**, **E**) SHAP values generated by evaluating the trained CPTAC gene copy number models on a random subset of the respective test set ($n = 300$). Negative SHAP values depict a higher contribution of a model's factor towards predicting an observation as *Buffered* relative to the baseline prediction. Each dot represents a gene in a sample. Color represents the min-max-scaled value of the factor used for prediction. Spearman's ρ indicates the trend between SHAP value and feature value. SHAP values were mean-centered within each factor.

© The Author(s)

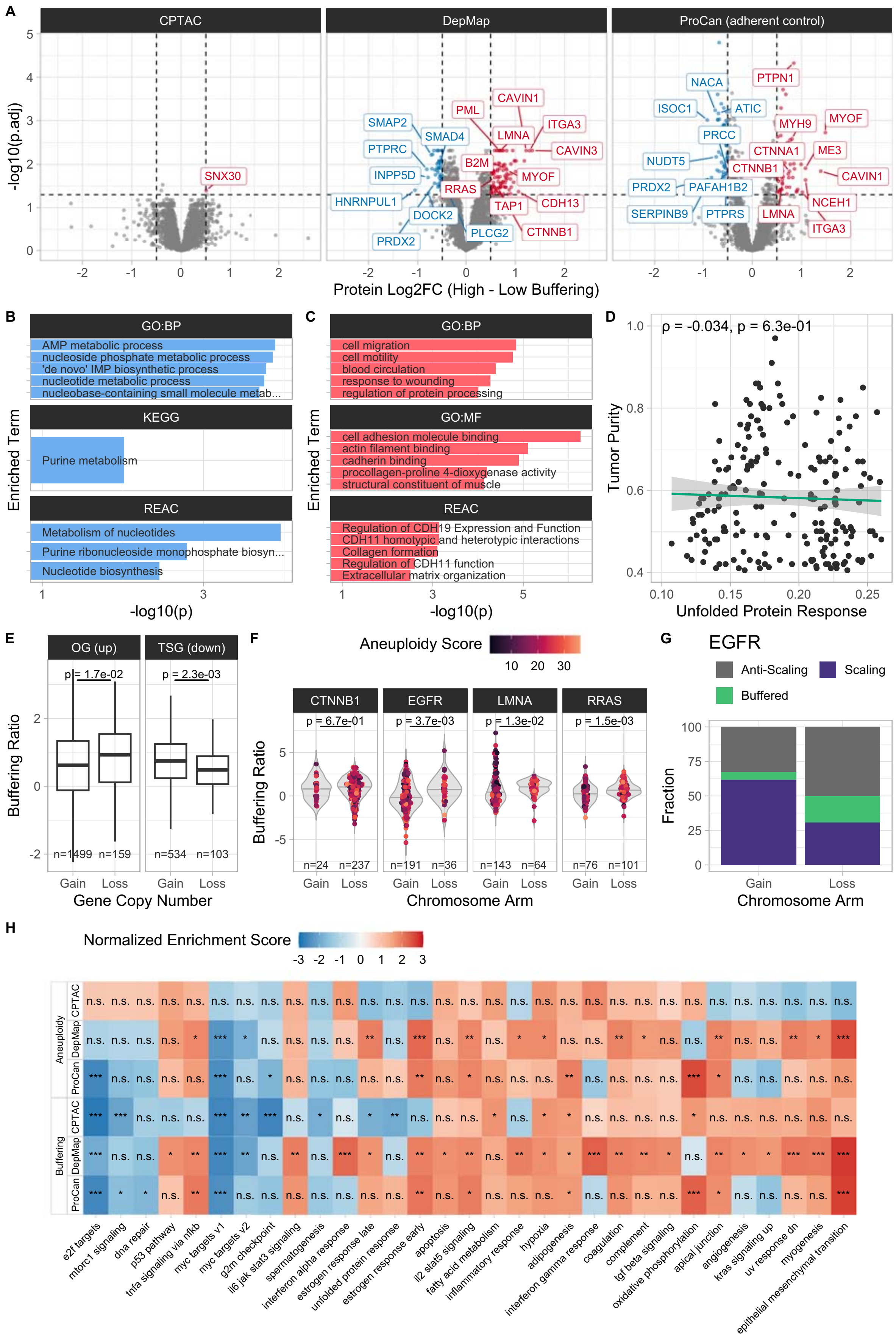

Figure EV5. Enrichment patterns suggest oncogenic effects of protein buffering.

(**A**) Volcano plots showing log2 fold change between high (>80% quantile of sample buffering ratios) and low (<20% quantile) buffering cell lines and tumor samples in DepMap ($n = 71$ per group), CPTAC ($n = 52$ per group), and adherent control datasets ($n = 82$ high buffering, $n = 51$ low buffering; Student's *t* test, Benjamini–Hochberg adjusted *P* values, significance thresholds: $|Log2FC| > 0.5$, $P_{adj} < 0.05$). (**B**, **C**) Overrepresentation analysis (ORA) of genes that are significantly down- (**B**) and upregulated (**C**) in highly buffering cell lines with adherent growth patterns (ProCan, adherent control; hypergeometric test using g:Profiler). (**D**) Correlation between tumor purity and single-sample enrichment scores for the *Unfolded Protein Response* HALLMARK gene set using CPTAC tumor sample proteome data (Spearman's ρ). (**E**) Buffering ratio distribution of oncogenes (OG) significantly upregulated in high buffering cell lines and of downregulated tumor suppressor genes (TSG) upon gene copy number gain and loss (ProCan, all cell lines). Boxes represent the interquartile range (IQR) with the central line indicating the median. The whiskers extend to the data points within 1.5×IQR. (**F**) Buffering ratio distribution of selected oncogenes upon gene copy number gain and loss (ProCan). (**G**) Categorical distribution of buffering classes for EGFR upon gain and loss of chromosome arm 7p (ProCan). (**H**) Normalized enrichment scores of MSigDB HALLMARK gene sets for pan-cancer datasets (DepMap, ProCan, CPTAC). Enrichment scores have been generated by comparing high against low-buffering samples and comparing high against low-aneuploid samples (>80% and <20% quantiles of sample buffering ratio and aneuploidy score estimates). Significance levels of Benjamini–Hochberg adjusted *P* values generated using fGSEA: $*P_{adj} < 0.01$, $**P_{adj} < 0.001$, $***P_{adj} < 0.0001$. Removed gene sets with no significant enrichment scores. (**E**, **F**) *P* values were determined using a two-tailed Wilcoxon rank-sum (Mann–Whitney *U*) test.

 © The Author(s)

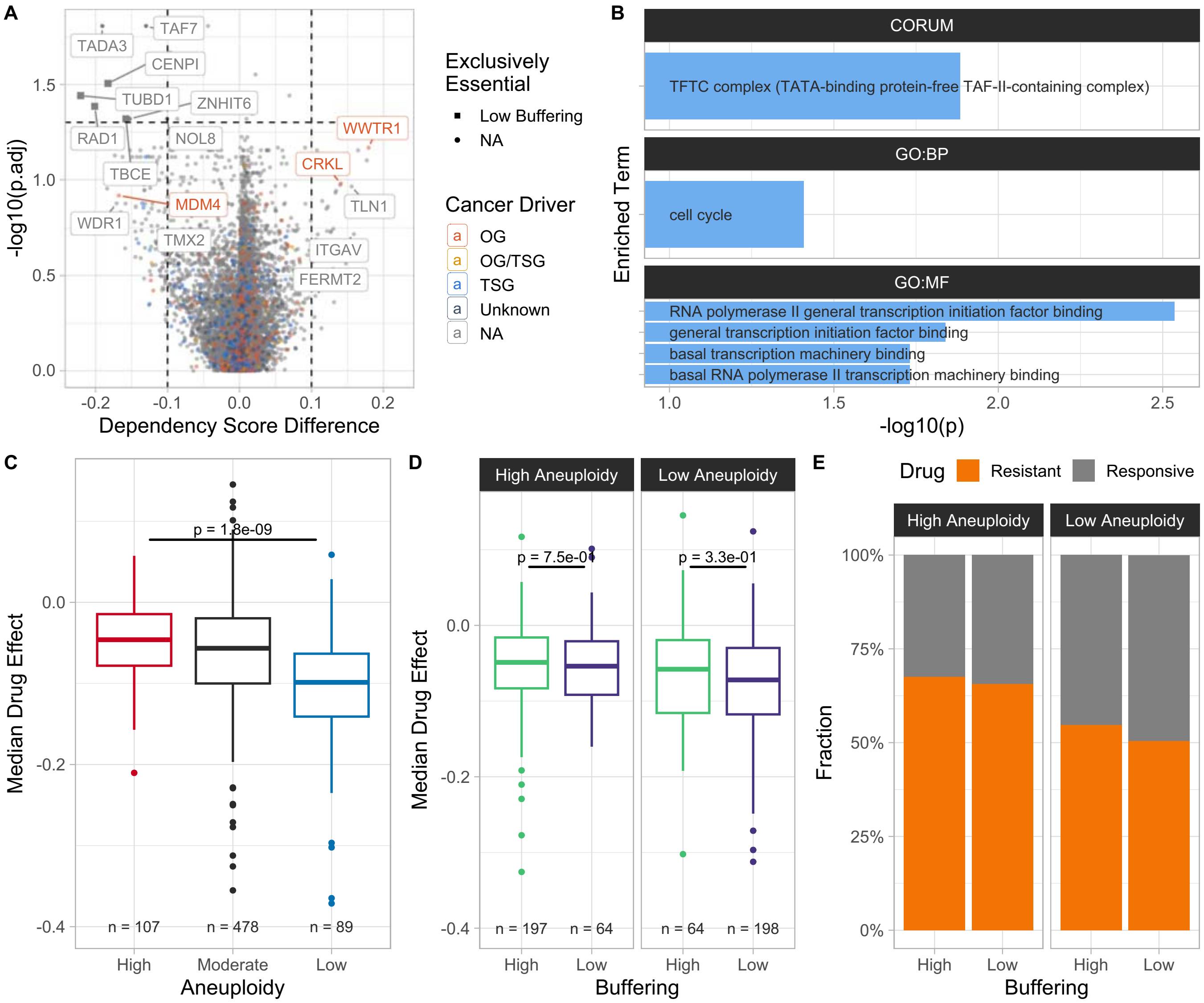

Figure EV6. Adherent and aneuploid controls for CRISPR knock-out and drug sensitivity analyses.

(**A**) Volcano plot showing the difference of the CRISPR-KO dependency score between high (>80% quantile of sample BR MNRs, $n = 90$) and low (<20% quantile, $n = 67$) buffering cell lines plotted against Benjamini–Hochberg adjusted Wilcoxon rank-sum test *P* values. Cell lines without adherent growth patterns were removed prior to the analysis. Genes were highlighted if they were significant in the adherent control or among the top 10 significant genes in Fig. 6A. (**B**) Over-representation analysis (ORA) of genes with significantly reduced CRISPR-KO dependency scores in highly buffering cell lines with adherent growth patterns (hypergeometric test using g:Profiler). (**C**) Median drug effect on cell viability across drugs from the PRISM Repurposing dataset for cell lines high (>80% quantile of aneuploidy score), moderate (20%-80%), and low aneuploidy (<20%). (**D**) Median drug effect for high (>50% sample BR MNR) and low-buffering cell lines, separated by median aneuploidy score. (**E**) Categorical distribution of drug-resistant (>50% median drug effect) and responsive cells between high (>50% sample BR MNR) and low-buffering cell lines, separated by median aneuploidy score. (**C**, **D**) Boxes represent the interquartile range (IQR) with the central line indicating the median. The whiskers extend to the data points within 1.5×IQR. *P* values were determined using a two-tailed Wilcoxon rank-sum (Mann–Whitney *U*) test.

© The Author(s)

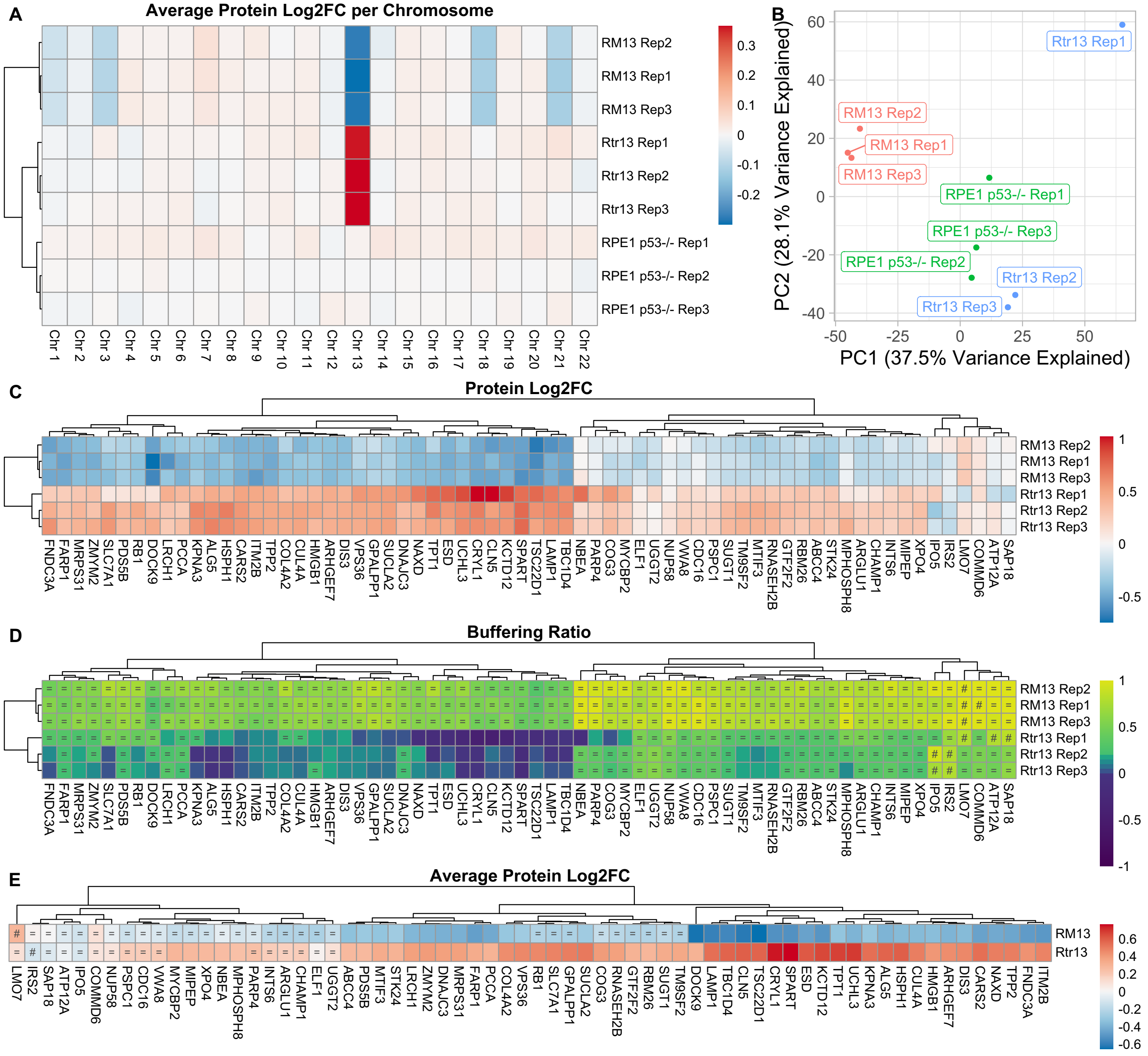

Figure EV7. Proteome and protein buffering analysis of RPE-1 cell lines with engineered aneuploidies on chromosome 13 (P0211).

(**A**) Mean log2 fold change of protein abundance per chromosome between cell line replicates and disomic baseline (median protein abundance of unaltered RPE-1 replicates per protein). (**B**) Principal components analysis (PCA) of P0211 replicates after normalization (first two principal components are displayed). (**C**) Protein abundance log2 fold change of proteins encoded on chromosome 13 between aneuploid replicates and disomic baseline. (**D**) Chromosome arm copy number-derived buffering ratios (BR) of proteins encoded on chromosome 13 in aneuploid replicates. Proteins are classified as *Buffered* (=) and *Anti-Scaling* (#) using BR-based thresholds (see "Methods"). (**E**) Mean protein abundance log2 fold-changes of proteins encoded on chromosome 13 between aneuploid and non-aneuploid cell lines. Proteins were classified as *Buffered* (=) and *Anti-Scaling* (#) using Log2FC-based thresholds (see "Methods").

 © The Author(s)
